# Dental Implants After Head and Neck Radiotherapy: From Chronological Waiting Rules to Implant-Bed-Specific Risk Stratification

**DOI:** 10.3390/healthcare14182889

**Published:** 2026-09-08

**Authors:** Erkan Topkan, Efsun Somay, Sibel Bascil, Ugur Selek

**Affiliations:** 1 Department of Radiation Oncology, Faculty of Medicine, Baskent University, 01230 Adana, Turkey; docdretopkan@gmail.com; 2 Department of Oral and Maxillofacial Surgery, Faculty of Dentistry, Baskent University, 01230 Adana, Turkey; 3 Department of Periodontology, Faculty of Dentistry, Baskent University, 01230 Adana, Turkey; bascil5@yahoo.com; 4 Department of Radiation Oncology, School of Medicine, Koc University, 34450 Istanbul, Turkey; ugurselek@yahoo.com

**Keywords:** dental implants, head and neck cancer, radiotherapy, implant-bed dosimetry, implant survival, risk stratification

## Abstract

**Highlights:**

**What are the main findings?**

- Implant-bed-specific radiation dose predicts implant outcomes more meaningfully than arbitrary post-radiotherapy waiting periods.
- Successful implant rehabilitation depends on the interaction of dosimetric, biological, prosthetic, and patient-related factors.

**What are the implications of the main findings?**

- Clinical decisions should shift from fixed waiting periods to individualized risk assessment based on implant-site characteristics.
- Future research should prioritize implant-site dosimetry and standardized risk-stratification models.

**Abstract:**

Dental implant-supported rehabilitation is a key component of functional survivorship care for patients with head and neck cancer (HNC) treated with radiotherapy (RT). Despite its importance, implant outcomes in this population are frequently interpreted through simplified paradigms such as irradiated versus non-irradiated status, prescribed tumor dose, pre-RT versus post-RT placement, and arbitrary post-RT waiting intervals. This narrative review proposes a radiobiology-informed, implant-bed-specific framework for assessing implant viability following HNC RT. The biologically relevant exposure is best defined as the localized radiation dose delivered to the implant bed, considered in conjunction with fractionation, equivalent dose in 2-Gy fractions, recipient substrate, soft-tissue condition, prosthetic loading, systemic host factors, and late toxicity risk. Distinctions among early osseointegration, implant survival, implant success, prosthetic rehabilitation, and patient-centered functional benefit are essential, as retained implants do not necessarily equate to durable or meaningful rehabilitation. Post-RT timing should be understood as a contextual variable rather than a universal chronological determinant, since longer intervals do not inherently indicate progressive biological recovery and may be confounded by survivor and clinician selection. Future research should prioritize implant-bed-specific dosimetry, continuous modeling of dose and timing, standardized outcome measures, cluster-adjusted statistical approaches, time-to-event analyses, and explicit reporting of osteoradionecrosis as a distinct implant-relevant late toxicity endpoint. Shifting from chronological waiting rules to implant-bed-specific risk stratification may enhance the safety, interpretability, and clinical relevance of dental implant rehabilitation following HNC RT.

## 1. Introduction

Dental implant-supported rehabilitation has become an important component of functional survivorship care for patients treated for head and neck cancer (HNC) [1]. Advances in surgery, reconstruction, radiotherapy (RT), systemic therapy, and supportive care have improved disease control and prolonged survival, increasing the importance of durable restoration of mastication, speech, swallowing, oral competence, facial aesthetics, and quality of life [1]. Implant-supported prosthetic rehabilitation may provide substantial functional and psychosocial benefits, particularly for patients with mandibular or maxillary defects, edentulism, compromised dentition, or complex post-treatment anatomy. However, implantation after HNC therapy occurs within a biologically altered environment in which host tissue quality, regenerative capacity, and long-term integration require particular consideration.

Radiotherapy, whether delivered definitively or as part of multimodality treatment, produces dose-dependent injury to bone, mucosa, microvasculature, salivary glands, and surrounding soft tissues [2,3,4,5]. Relevant consequences include osteoblast depletion, impaired bone remodeling, endothelial dysfunction, hypovascularity, hypocellularity, fibrosis, mucosal fragility, xerostomia, infection susceptibility, and osteoradionecrosis (ORN) [2,3,4,5]. Implant osseointegration and long-term maintenance therefore cannot be understood solely as mechanical or prosthetic events; they reflect interactions among local radiation injury, residual tissue repair capacity, recipient-site characteristics, systemic host factors, prosthetic loading, and late toxicity risk.

Despite decades of clinical experience, the determinants of implant viability after HNC RT remain incompletely defined. Existing studies frequently interpret outcomes through simplified constructs such as irradiated versus non-irradiated status, prescribed radiation dose, pre- versus post-RT placement, and categorically defined post-treatment intervals [3,4]. Although clinically convenient, these variables may be biologically imprecise. The radiation exposure most relevant to an implant is not necessarily the prescribed tumor dose or a patient-level dose category, but the actual dose delivered to the implant bed [6,7,8]. Likewise, longer waiting after RT does not necessarily indicate progressive tissue recovery [9,10], because acute and subacute injury may evolve into persistent hypovascular, hypocellular, and fibroatrophic changes [5]. Historically useful treatment- and chronology-based classifications should therefore be reconsidered in light of contemporary RT planning and localized dosimetric assessment, which permit evaluation centered more directly on the implant bed [1,5,6,7,8,9,10].

These limitations also have methodological implications. A favorable outcome after implantation several years following RT may reflect not only the effect of elapsed time but also survivor and clinician selection, because patients remaining recurrence-free, medically fit, and free from severe treatment-related complications are more likely to undergo elective rehabilitation. Apparent timing effects may also be confounded by implant-bed dose, reconstructive anatomy, oral hygiene, soft-tissue integrity, prosthetic design, and clinical selection. In addition, many studies analyze multiple implants within individual patients without adequately accounting for intrapatient clustering, despite their shared oncologic, dosimetric, systemic, reconstructive, and follow-up characteristics. Short follow-up may capture early osseointegration while missing late implant loss, peri-implant bone degradation, prosthetic failure, or ORN, and exclusion of patients with recurrence or severe complications may further accentuate survivor-selection effects. Together, these issues may distort estimates of implant viability and the apparent influence of timing and other candidate determinants.

This review seeks to reconceptualize dental implant rehabilitation after HNC RT by moving beyond a predominantly timing-centered perspective toward an implant-bed-specific, radiobiology-informed, and methodologically robust framework. We critically examine how localized radiation dose, temporal tissue injury, recipient-site characteristics, peri-implant soft-tissue quality, prosthetic loading, ORN risk, and statistical design shape implant outcomes. We further propose a risk-adapted approach for future research and clinical decision-making that prioritizes implant-bed dosimetry, continuous modeling of timing, appropriate handling of clustered and time-to-event outcomes, and explicit recognition of survivor-selection effects.

## 2. Review Design, Scope, and Literature Search Strategy

This article was conceived as a critical narrative review supported by a conceptual framework. Rather than undertaking a formal quantitative synthesis of implant survival rates, we aimed to appraise how dental implant outcomes after head and neck RT have been conceptualized, measured, analyzed, and interpreted. Particular attention was given to implant-bed-specific dosimetry, radiobiological tissue response, implant-placement timing, recipient bone and soft-tissue characteristics, prosthetic loading, ORN risk, and methodological limitations.

The review focused on studies of dental implant placement or implant-supported prosthetic rehabilitation in patients with HNC treated with RT, alone or as part of multimodality therapy. Studies were considered when they reported implant outcomes in relation to RT exposure, timing, radiation dose, recipient bone or reconstructive characteristics, peri-implant bone loss, implant survival, prosthetic rehabilitation, or ORN. Primary or pre-RT implant placement was included only when necessary to provide comparative context for post-RT outcomes; detailed evaluation of primary implant-reconstruction protocols was outside the principal scope.

A structured literature search was conducted in PubMed/MEDLINE, Scopus, Web of Science, and Google Scholar from database inception through 15 July 2026. Search terms included combinations of “head and neck cancer,” “radiotherapy,” “dental implant,” “implant survival,” “implant failure,” “osseointegration,” “implant-bed dose,” “site-specific dose,” “mandible dose,” “maxilla dose,” “vascularized bone flap,” “prosthetic rehabilitation,” “osteoradionecrosis,” and “timing.” Reference lists of relevant reviews, meta-analyses, and cohort studies were also screened.

Evidence was synthesized narratively and interpreted according to methodological rigor, including outcome definition, follow-up duration, granularity of localized dosimetry, distinction between patient- and implant-level outcomes, handling of multiple implants per patient, and adjustment for clinically relevant confounders.

## 3. Defining Implant Success After Head and Neck Radiotherapy

A principal challenge in appraising the literature on dental implants after head and neck RT is the inconsistent use of outcome terminology. Terms such as implant success, implant survival, implant viability, osseointegration, and prosthetic rehabilitation are frequently used interchangeably, although they refer to distinct clinical states [11,12,13]. This imprecision is especially consequential in irradiated patients, in whom early implant stability does not necessarily predict durable peri-implant bone health, sustained prosthetic function, or freedom from delayed complications such as ORN [2,3,4,5,14].

In conventional implant dentistry, implant success has traditionally been defined by the absence of mobility, pain, infection, peri-implant radiolucency, and excessive progressive bone loss [11,12]. Although these criteria remain clinically useful, their application in the post-RT HNC population requires caution. Irradiated bone is characterized by compromised vascularity, impaired remodeling potential, diminished cellular reserve, and increased susceptibility to soft-tissue breakdown and infection [2,3,4,5]. Consequently, an implant that appears clinically stable during early healing may remain vulnerable to delayed marginal bone loss, peri-implant soft-tissue complications, prosthetic failure, or late implant loss [2,3,4,6,7]. In this context, early osseointegration should be regarded as a necessary, yet insufficient, endpoint.

A hierarchy of outcome measures is therefore required. Early osseointegration refers to the initial biological anchorage of the implant, typically assessed within the first several months after placement. Implant survival refers to the continued presence of the implant in situ, regardless of functional status, complication profile, or prosthetic utility [12,13]. Implant success requires a more rigorous assessment, including absence of clinical symptoms, infection, mobility, progressive bone loss, and peri-implant pathology [11,12]. Prosthetic rehabilitation success extends beyond the implant itself and reflects durable, functional, maintainable, and patient-centered oral rehabilitation [1,13,15]. Long-term implant viability in irradiated HNC survivors should be conceptualized as a composite outcome integrating implant retention, peri-implant bone stability, soft-tissue health, prosthetic function, hygiene accessibility, patient-reported benefit, and freedom from late toxicity [1,2,14,15].

This distinction is not merely semantic; it directly influences the interpretation of the study. Studies with short follow-up periods, commonly three to six months, may provide information on recipient-site acceptability and early osseointegration but cannot adequately assess long-term viability in irradiated tissues [2,3,4,9]. Late implant loss, marginal bone degradation, prosthetic non-use, chronic peri-implant infection, and ORN may appear months or years after initial integration [2,3,4,5,14]. Moreover, studies that report implant survival without data on prosthetic loading, functional rehabilitation, or late complications may overstate the clinical value of treatment [1,13,15].

The analytic unit of outcome assessment is also consequential. Patient-level success and implant-level success answer different clinical questions. Implant-level reporting quantifies the proportion of retained fixtures, whereas patient-level reporting better captures whether the individual achieves meaningful functional rehabilitation [13,15]. Loss of one strategically positioned implant may compromise an entire prosthetic plan despite the survival of other fixtures. Conversely, retention of multiple implants may confer limited benefit if the patient cannot tolerate, maintain, or use the prosthesis effectively because of xerostomia, trismus, soft-tissue fibrosis, or recurrent infection [1,14,15]. Future studies should therefore report both implant-level and patient-level outcomes while explicitly distinguishing biological integration from prosthetic and functional success.

For HNC survivors treated with RT, a tiered outcome framework is likely to provide greater clinical and scientific insight than reliance on a single binary endpoint. At minimum, studies should report early osseointegration, implant retention, peri-implant bone stability, prosthetic loading and use, late implant loss, ORN, and patient-centered functional endpoints [1,13,14,15]. Such granularity would clarify whether a given intervention truly advances long-term rehabilitation or merely improves short-term implant retention. This distinction is essential for robust conclusions regarding the influence of RT timing, implant-bed dosimetry, recipient substrate, or post-treatment interval on implant-related outcomes. To operationalize this distinction, the proposed outcome hierarchy summarized in Table 1 separates early biological integration from implant retention, implant success, prosthetic rehabilitation, patient-centered functional benefit, and implant-relevant late toxicity, thereby providing a more comprehensive framework for evaluating rehabilitation outcomes after HNC RT.

## 4. Radiobiology of the Irradiated Implant Bed

The irradiated implant bed represents a biologically altered tissue environment in which osseointegration, peri-implant bone preservation, soft-tissue stability, and resistance to microbial challenge may be compromised. Successful implantation after head and neck RT depends on adequate residual vascularity, cellularity, remodeling potential, mucosal integrity, and tolerance to prosthetic loading. Ionizing radiation exerts dose-dependent effects on bone, endothelium, mucosa, salivary glands, connective tissue, and immune-regulatory pathways [2,3,4,5,14,16,17,18]. Importantly, this injury is spatially heterogeneous and influenced by the dose delivered to the implant-bearing region [5,6,7,8], underscoring the limitations of classifying implant-bed biology simply as irradiated versus non-irradiated.

At the osseous level, radiation can deplete or impair osteoblasts and osteoprogenitor cells, reduce osteocyte viability, and attenuate physiological remodeling [5,14,16,17,18]. Irradiated bone may therefore retain structural continuity while losing part of its capacity to respond to surgical trauma, microbial challenge, and biomechanical loading. Thus, structural preservation of the mandible or maxilla does not necessarily imply preserved regenerative potential. In higher-dose regions, reduced cellular reserve and remodeling capacity may manifest as delayed osseointegration, marginal bone loss, peri-implant infection, mobility, or late implant failure [2,3,4,5,6,7,8,14].

Radiation-induced endothelial injury may produce capillary rarefaction, luminal narrowing, thrombosis, perivascular fibrosis, reduced perfusion, and progressive tissue hypoxia [14,16,17,18], thereby impairing oxygen delivery and wound healing. The classic hypovascular–hypocellular–hypoxic model remains clinically instructive for understanding impaired healing and vulnerability to local trauma [16], although it does not fully characterize irradiated tissue biology. The fibroatrophic paradigm further emphasizes chronic oxidative stress, endothelial dysfunction, aberrant inflammatory signaling, extracellular matrix deposition, and transforming growth factor-β–mediated fibrosis [17,18]. Together, these mechanisms indicate that the irradiated implant bed may be constrained by both ischemic-cellular depletion and maladaptive fibroinflammatory remodeling.

**Figure 1:**
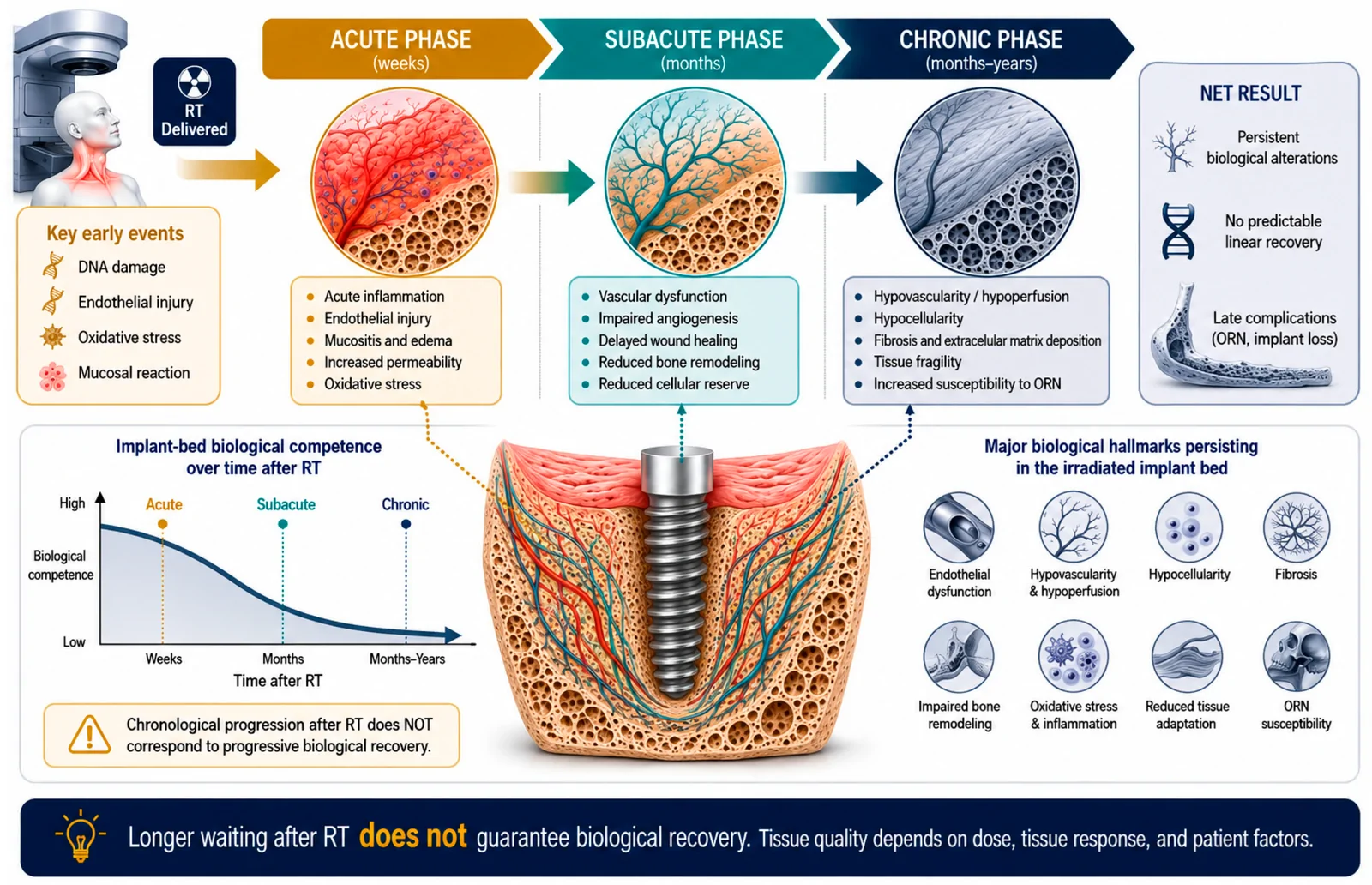
Temporal and Biological Evolution of the Irradiated Implant Bed after Head and Neck Radiotherapy. Schematic representation of the temporal evolution of radiation-associated tissue injury relevant to implant rehabilitation. Acute injury is characterized by inflammation, endothelial damage, oxidative stress, and mucosal reactions; the subacute phase may involve persistent vascular dysfunction, impaired angiogenesis and wound healing, reduced bone remodeling, and declining cellular reserve; and chronic injury may be characterized by hypovascularity, hypocellularity, fibrosis, tissue fragility, and increased susceptibility to osteoradionecrosis. The central illustration summarizes persistent biological alterations within the irradiated implant bed. The temporal curve is conceptual and is intended to emphasize that increasing time after RT does not necessarily correspond to progressive biological recovery; it should not be interpreted as a quantitative longitudinal trajectory. Implant-bed competence remains dependent on localized radiation exposure, tissue response, recipient substrate, and patient-related factors.

The temporal evolution of these alterations is central to interpreting implant timing. Although acute mucosal reactions may resolve within weeks, visible epithelial restitution does not necessarily indicate recovery of underlying osseous, stromal, or microvascular compartments [5,14,17,18]. Persistent endothelial dysfunction and impaired tissue repair may progress toward chronic hypovascular, hypocellular, hypoxic, and fibrotic states [16,17,18]. Consequently, a longer post-RT interval should not automatically be equated with progressive biological recovery; later tissue states may remain characterized by fibrosis, reduced vascular reserve, impaired remodeling, and diminished tolerance to surgical or prosthetic stress [5,17,18]. This trajectory challenges the assumption that extended waiting is inherently beneficial (Figure 1).

Soft-tissue and salivary gland injury further modify implant risk. Xerostomia, mucosal thinning, fibrosis, trismus, dysbiosis, and impaired hygiene access may increase peri-implant inflammation and compromise prosthetic maintenance [1,5,14,15,18]. Sustained implant viability also depends on an effective peri-implant mucosal seal, adequate soft-tissue coverage, salivary function, prosthetic design, and maintainable oral hygiene [1,14,15]. Local infection, dysbiosis, and peri-implant inflammation may further challenge healing when vascularity, host defense, mucosal integrity, and bone remodeling are compromised [6,14,15,18]. Thus, late peri-implant complications should be viewed as interactions between osseous radiation injury and the surrounding soft-tissue and microbial environment rather than as bone- or implant-surface phenomena alone.

ORN represents the most severe manifestation of this biological vulnerability. Implant failure and ORN are distinct endpoints and should not be conflated, although both may arise in a compromised tissue bed characterized by reduced vascularity, hypoxia, impaired remodeling, infection susceptibility, and fibroinflammatory change [14,16,17,18]. Implant placement, peri-implant infection, prosthetic trauma, or implant loss may act as local stressors, particularly when the implant bed has received substantial radiation exposure [5,6,7,8,14]. ORN should therefore be reported separately, especially when temporally or anatomically related to implant placement, peri-implant infection, or implant loss [14,19]. Table 2 summarizes the major tissue-level effects of RT and their implications for implant rehabilitation.

Taken together, the radiobiology of the irradiated implant bed supports three principles. First, implant outcomes are site-specific because radiation injury is spatially heterogeneous and dose-dependent [5,6,7,8]. Second, implant timing should be interpreted in relation to tissue phase, localized dose history, and candidate selection rather than as a simple chronological variable [9,10,17,18]. Third, long-term viability depends not only on implant fixation but also on vascular reserve, bone remodeling capacity, peri-implant soft-tissue integrity, prosthetic maintainability, and freedom from severe late toxicity [1,5,14,15,19]. These principles provide the biological rationale for prioritizing implant-bed-specific dosimetry, nonlinear timing assessment, and clinically meaningful long-term endpoints in future implant rehabilitation studies.

## 5. Implant-Bed-Specific Dosimetry: The Missing Determinant

A major limitation in the literature addressing dental implant outcomes after head and neck RT is the frequent reliance on non-localized measures of radiation exposure. Many studies categorize patients according to whether they received RT, whether implants were placed before or after RT, or whether the prescribed dose exceeded broad thresholds such as 50, 60, or 66 Gy. Although such classifications are clinically convenient, they do not necessarily capture the biological exposure experienced by the implant-bearing bone. The most anatomically relevant radiation exposure for osseointegration, peri-implant bone preservation, and late implant survival is the dose delivered to the implant bed itself, rather than the prescribed tumor dose or a general patient-level treatment category [6,7,8].

This distinction is particularly important in head and neck RT, where spatial heterogeneity of dose distribution is inherent. Three-dimensional conformal RT, intensity-modulated RT, and volumetric modulated arc therapy can create steep dose gradients across the mandible, maxilla, reconstructed bone, and adjacent soft tissues. As a result, two patients with identical prescribed tumor doses may experience markedly different implant-bed exposures depending on tumor subsite, laterality, target-volume extent and nodal irradiation, boost volume, implant location, beam arrangement, dose constraints, and reconstructive anatomy [5,6,7,8]. Although tumor stage may correlate with the extent and complexity of oncologic treatment, it is an upstream clinical descriptor and should not be used as a surrogate for the localized radiation exposure of the prospective implant bed. Similarly, implants placed in distinct anatomical regions within the same patient may receive different radiation doses. Thus, binary classifications such as “irradiated” versus “non-irradiated,” or broad categorical comparisons such as <60 Gy versus >60 Gy, risk substantial misclassification of exposure.

A further radiobiological refinement concerns the distinction between physical dose and biologically effective dose. Even when the physical dose delivered to the implant bed is known, its biological effect may vary with dose per fraction, total number of fractions, fractionation schedule, treatment modality, and any contribution from boost irradiation or reirradiation. Thus, identical implant-bed physical doses may correspond to different equivalent doses in 2-Gy fractions (EQD2), particularly for late-responding tissues such as bone, microvasculature, and connective tissue [20,21]. This distinction may partly explain heterogeneity in reported dose thresholds across implant studies, particularly when cohorts include different fractionation schedules, boost contributions, or reirradiation histories. Future studies should therefore specify not only implant-bed mean dose (Dmean) and maximum dose (Dmax), but also fraction size, total number of fractions, and, when appropriate, EQD2-based implant-bed dose estimates.

Implant-bed-specific dosimetry aligns quantified radiation exposure with the anatomical site of osseointegration, permits implant-level rather than patient-level risk assessment, and enables evaluation of dose–response relationships and interactions with timing, recipient substrate, prosthetic loading, and fractionation. When anticipated implant sites can be identified during RT planning, localized exposure may also be documented and, when oncologically feasible, considered in subsequent rehabilitation planning.

**Figure 2:**
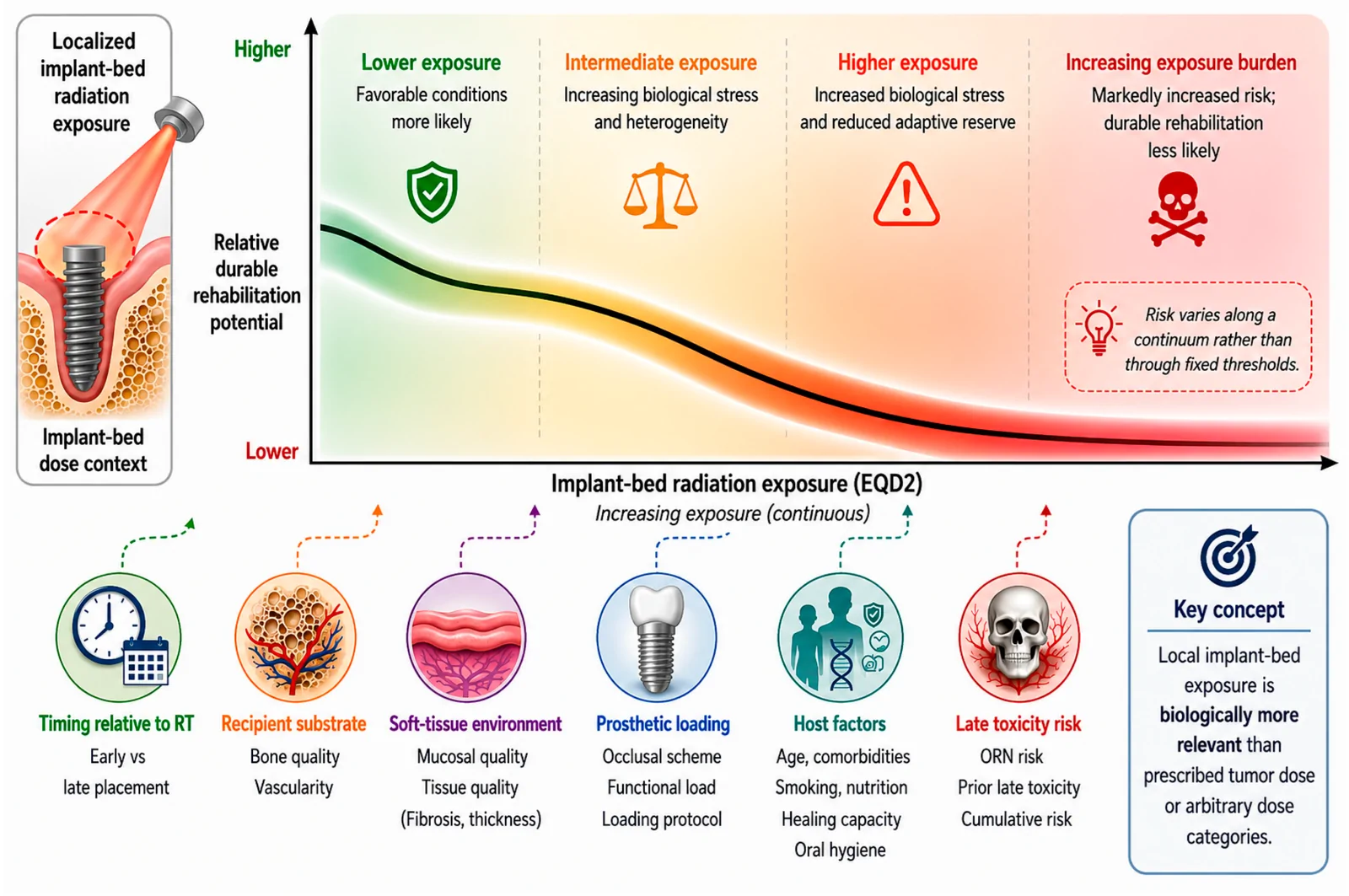
Conceptual Relationship between Implant-Bed Radiation Exposure and Durable Rehabilitation Potential. The figure illustrates the central premise of the review: as localized radiation exposure to the prospective implant bed increases, biological reserve and the potential for durable implant rehabilitation may progressively decline, although the relationship is modified by recipient substrate, soft-tissue quality, timing, prosthetic loading, host factors, and late-toxicity risk. The model represents a graded risk continuum rather than fixed biological thresholds. The trajectory is conceptual and should not be interpreted as a validated quantitative dose–response curve or predictive model. Accordingly, implant-bed dose should be interpreted together with tissue and clinical context rather than used as a deterministic eligibility criterion.

Available implant-level dosimetric evidence, although still limited, demonstrates a broadly consistent dose–response pattern. Wolf et al. reported an inverse relationship between radiation dose at the implant site and implant success, with an implant-specific dose > 50 Gy emerging as a strong predictor of implant failure [6]. Lee et al. similarly identified implant-site Dmean as an independent prognostic factor for implant survival and reported no implant failures when Dmean was <38 Gy [7]. In patients with implants present before RT, Li et al. demonstrated significantly poorer radiographic success and greater peri-implant bone resorption when the implant-bed-specific dose exceeded 40 Gy [8]. In a subsequent cohort, Alberga et al. reported peri-implant bone loss in implants receiving Dmean > 40 Gy, whereas implant loss occurred only among implants receiving Dmean > 50 Gy [22]. Collectively, these observations suggest clinically useful, albeit approximate, dose zones: implant-bed exposures below approximately 38–40 Gy appear comparatively favorable; exposures between approximately 40 and 50 Gy may represent an intermediate-risk range in which peri-implant bone changes become increasingly relevant; and exposures > 50 Gy appear to be associated with a substantially greater risk of implant failure. These ranges should not, however, be interpreted as validated biological thresholds. They arise from relatively small, heterogeneous observational cohorts that differ in implant timing, recipient substrate, outcome definition, follow-up, radiation technique, and statistical approach, and they have not been prospectively or externally validated as deterministic cutoff values. Accordingly, their principal value lies in risk stratification rather than deterministic prediction (Figure 2).

Importantly, dose levels associated with implant failure should not be conflated with ORN thresholds. The dosimetric literature on mandibular ORN has generally evaluated whole-mandible or regional dose–volume relationships rather than implant-bed-specific Dmean. In a case-matched analysis of 68 ORN cases and 131 controls treated with intensity-modulated RT, the MD Anderson Head and Neck Cancer Symptom Working Group reported a higher mean mandibular dose among patients with ORN (48.1 versus 43.6 Gy), while dose–volume parameters across a broad intermediate- to high-dose range (V35–V73) were also significantly greater in the ORN group. Mandibular V44 < 42% and V58 < 25% were proposed as reasonable planning constraints in that cohort [23]. These findings reinforce the view that ORN risk is related to the volume and spatial distribution of mandibular irradiation rather than to a single, universally applicable point-dose threshold. Accordingly, exposures approaching or exceeding 60 Gy should be regarded as representing an increasingly unfavorable dosimetric context, particularly when accompanied by mandibular location, impaired soft-tissue coverage, xerostomia, infection, prior surgery, or other local risk factors, rather than as a universal ORN threshold [5,6,7,8,14,19]. Thus, the available evidence better supports a graded dose–response continuum modified by anatomical, biological, and clinical factors than a binary safe-versus-unsafe radiation dose. The clinical value of the approximately 38–40-, 40–50-, and >50-Gy ranges is therefore in risk stratification and counseling, not in the deterministic selection or exclusion of candidates for implant rehabilitation.

Localized radiation dose should nevertheless be interpreted within its anatomical and biological context rather than as an isolated determinant. Recipient substrate, soft-tissue integrity, systemic factors, prosthetic loading, and fractionation may all modify the biological consequences of a given implant-bed exposure.

Future studies should therefore report radiation exposure with greater anatomical, dosimetric, and radiobiological precision. At minimum, investigators should specify whether the reported dose corresponds to the prescribed tumor dose, mandibular or maxillary dose, reconstructed-bone dose, or implant-bed-specific dose. When treatment plans are available, extract implant-region dose metrics such as Dmean, Dmax, and clinically relevant dose-volume parameters at the planned or actual implant site. Studies involving multiple implants should report the dose separately for each implant whenever feasible, because implants within the same patient may occupy different dose regions. In addition, report fraction size, total number of fractions, boost contribution, and reirradiation status, and consider EQD2 estimates when fractionation differs across cohorts. Such reporting would enable more meaningful dose–response modeling, clarify interactions between dose and timing, and reduce exposure misclassification, which currently limits interpretation of many retrospective studies.

In clinical practice, implant-bed-specific dosimetry can be incorporated prospectively when anticipated implant sites are known or reconstructed retrospectively from the original RT plan after treatment. This information can then inform multidisciplinary rehabilitation planning, including implant selection, surgical sequencing, loading strategy, follow-up intensity, and patient counseling, while oncologic target coverage remains the overriding priority.

Taken together, the available evidence and radiobiological rationale indicate that implant-bed dose should be considered a central determinant in dental implant rehabilitation after head and neck RT. Timing, recipient substrate, and prosthetic variables remain important, but their interpretation is incomplete without knowledge of localized radiation exposure. Moving from prescribed-dose categories toward implant-bed-specific, fractionation-aware dosimetry is therefore essential for developing a risk-adapted framework for implant placement, maintenance, and long-term rehabilitation in irradiated HNC survivors.

## 6. Pre-RT Versus Post-RT Implant Placement: Timing as a Modifier, Not a Stand-Alone Determinant

The distinction between pre- and post-RT implant placement has been a focal topic in implant-supported rehabilitation after HNC. Pre-RT placement permits implant insertion and initial osseointegration before radiation-induced vascular, cellular, and fibroatrophic changes develop, whereas post-RT placement occurs in a tissue bed that may already be hypovascular, hypocellular, hypoxic, fibrotic, and less capable of remodeling [2,3,4,5,16,17,18]. Several studies and systematic reviews report higher implant survival when implants are placed before, or concurrently with, ablative or reconstructive surgery and before adjuvant RT than with secondary post-RT placement [2,3,4,9,10,15,24,25,26].

Terminology requires care. In reconstructive HNC literature, “immediate” or “primary” implantation generally denotes placement during ablative or reconstructive surgery, whereas “delayed” or “secondary” implantation refers to placement after the initial surgical procedure [25,26]. Secondary implantation after RT should not be conflated with the separate question of how long to wait after RT, which is addressed in Section 7.

The apparent advantage of pre-RT placement should not be interpreted as evidence that timing alone determines implant outcome. Pre-RT implants may benefit from initial placement in non-irradiated or minimally injured bone, but long-term outcomes remain influenced by the dose subsequently delivered to the implant bed, fractionation, peri-implant soft-tissue status, prosthetic loading, and late radiation effects [5,6,7,8,20,21]. Conversely, post-RT implants in low-dose regions with preserved mucosal coverage, adequate vascularity, favorable hygiene access, and limited prosthetic stress may achieve satisfactory outcomes. Thus, pre-RT and post-RT categories are clinically informative but do not capture the full biological risk.

Primary or pre-RT implantation may reduce the need for later surgery in irradiated tissues, permit osseointegration before chronic radiation effects develop, and, in patients undergoing vascularized mandibular reconstruction, facilitate earlier prosthetic planning and rehabilitation [1,9,15,24,25]. Such placement requires multidisciplinary planning to ensure that implant sites are oncologically safe, surgically accessible, prosthetically appropriate, and compatible with RT planning [24,25]. Moreover, implants in regions subsequently receiving high-dose RT may remain vulnerable to impaired remodeling, marginal bone loss, soft-tissue complications, or late failure [6,7,8]. Pre-RT success therefore depends not only on timing but also on favorable local dosimetric, anatomical, and prosthetic conditions.

Post-RT placement remains an important option for patients who are not candidates for primary implantation, including those treated with definitive RT or chemoradiotherapy or those requiring delayed rehabilitation. In such patients, implantation can provide functional rehabilitation when decisions account for implant-bed dose, local tissue quality, anatomical site, systemic health, oral hygiene, xerostomia, trismus, prosthetic considerations, and ORN risk [1,5,9,14,15,19]. The central question is therefore whether the recipient site retains sufficient biological and mechanical capacity for long-term success, rather than timing relative to RT alone.

Comparisons between pre-RT and post-RT outcomes are also vulnerable to confounding and selection. Patients selected for primary placement may differ from those receiving implants after RT in tumor characteristics, resection and reconstruction, prognosis, dentition, general health, nutrition, smoking, and access to multidisciplinary care. Post-RT implant recipients also constitute a survivor cohort that has remained recurrence-free and fit for elective surgery. Apparent timing effects may therefore reflect a combination of biological differences, implant-bed dose, baseline characteristics, and clinical selection.

Future studies should not treat pre-RT versus post-RT placement as a self-sufficient explanatory variable. Timing should be reported alongside implant-site dose, EQD2 where relevant, recipient bone type, soft-tissue status, prosthetic loading, systemic risk factors, and follow-up. Interaction analyses may clarify whether timing effects differ by implant-bed dose, recipient substrate, anatomical site, or loading status, and whether apparent effects are independent of or modified by localized radiation exposure and tissue biology.

Taken together, pre-RT and post-RT placement remain clinically meaningful categories, but neither should be viewed as an independent determinant of implant success. Their relevance depends on the dosimetric, anatomical, reconstructive, and prosthetic context of the implant bed. This distinction is particularly important when considering post-RT waiting intervals, which should not be assumed to represent biological recovery simply because more time has elapsed.

## 7. The Post-RT Timing Fallacy: Why Longer Waiting May Not Mean Better Biology

The interval between RT completion and secondary implant placement is often considered a significant factor in dental rehabilitation following HNC. Numerous studies classify post-RT implantation according to specific chronological thresholds, such as placement before or after 6, 12, 18, or 24 months, while others compare mean or median intervals between successful and unsuccessful implants [9,10,27,28]. Although such comparisons are straightforward to report, they may encourage the assumption that longer waiting periods after RT inherently improve implant-bed quality and implant survival, a notion that requires critical evaluation.

Radiobiological principles suggest that post-RT tissue recovery does not follow a simple linear pattern in which each additional month leads to greater healing capacity. While acute mucosal reactions may resolve within weeks and early clinical stabilization may occur over the following months, the underlying bone, stromal, and microvascular compartments may continue to show endothelial dysfunction, impaired remodeling, hypoxia, oxidative stress, and fibroatrophic changes [5,16,17,18]. Once chronic hypovascular, hypocellular, hypoxic, and fibrotic remodeling is established, further waiting does not necessarily result in meaningful biological recovery. Instead, extended intervals after RT may reflect clinical stability within a persistently altered tissue environment, rather than restoration of baseline physiology [16,17,18].

This distinction is relevant when evaluating interval-based studies. For example, Sukumaran et al. reported that successful post-RT implants had a longer mean interval from RT completion than failed implants (approximately 54 versus 37 months, respectively) [27]. However, such differences in average timing do not, by themselves, define a biologically meaningful threshold. Any distribution of implantation intervals will yield central tendency values, and observed differences between outcome groups may reflect underlying clinical decision-making rather than a true recovery period. Without continuous modeling or biologically justified thresholds, mean or median interval comparisons may inadvertently transform descriptive observations into unsupported therapeutic guidance [9,10,29,30].

The post-RT interval is also susceptible to survivor-selection bias. Individuals who receive implants after longer waiting periods may not represent the broader population of patients treated with RT. These patients are often those who have remained recurrence-free, avoided severe early toxicity, maintained adequate nutritional and functional status, and retained access to specialized dental oncology care. In contrast, patients with early recurrence, severe ORN, persistent xerostomia, poor wound healing, severe trismus, cachexia, or significant comorbidities may not reach the stage of implant consideration. As a result, favorable outcomes in delayed post-RT implant cohorts may partly reflect the selection of healthier survivors, rather than a direct biological effect of prolonged waiting [9,10,31].

Clinical selection further complicates interpretation. In retrospective studies, implant timing is rarely random. Surgeons and prosthodontists may defer implantation in patients with poor mucosal quality, high-dose mandibular exposure, unstable oncologic status, or inadequate oral hygiene, while favoring earlier placement in those with more favorable tissue conditions. When analyzed retrospectively, these choices may introduce associations between timing and outcome that reflect clinical judgment, tissue selection, implant-bed dose, or patient fitness rather than the effect of time itself. This situation illustrates confounding by indication, in which timing serves in part as a marker of candidate suitability rather than a direct biological exposure [31,32].

The post-RT interval should also be interpreted in relation to implant-bed dose: prolonged waiting cannot be assumed to compensate for severe local radiation injury, whereas a shorter interval may remain clinically reasonable in a lower-dose site with favorable tissue conditions [5,6,7,8,16,17,18,20,21].

Xerostomia should be considered within this contextual assessment, but current evidence does not support using its severity to prescribe a specific post-RT waiting interval for implant placement [9,10]. Reduced salivary function may increase microbial burden, impair oral hygiene, promote peri-implant inflammation, compromise mucosal tolerance, and reduce the maintainability of implant-supported prostheses [1,5,14,15,18]. Severe or poorly controlled xerostomia may therefore make a patient a less favorable candidate for implantation at a given time and may justify optimization of oral care, salivary management, mucosal condition, and prosthetic planning before proceeding. However, delaying implantation by an arbitrary number of additional months cannot be assumed to reverse established salivary gland injury or to convert an unfavorable implant bed into a favorable one. Xerostomia severity should therefore be regarded as a modifier of overall candidacy, tissue and maintenance risk, and readiness for rehabilitation rather than as an independent chronological determinant of implant timing.

Future studies should therefore avoid treating post-RT timing as a crude categorical variable unless thresholds are clinically and biologically justified. Whenever sample size permits, model the interval from RT completion to implant placement continuously using restricted cubic splines, fractional polynomials, segmented regression, or other flexible approaches that can detect nonlinear associations [29,30]. Time-to-event analyses should also account for competing events such as recurrence, death, ORN, medical ineligibility, or failure to proceed to rehabilitation. Such methods would help distinguish whether timing exerts an independent effect, acts as a surrogate for patient selection, or interacts with implant-bed dose and tissue substrate.

Taken together, biological and methodological considerations challenge the assumption that longer waiting after RT is inherently beneficial. A more defensible interpretation is that post-RT timing may have a nonlinear and context-dependent relationship with implant viability: early placement may occur before adequate clinical stabilization, whereas prolonged delay may offer diminishing benefit or coincide with chronic fibroatrophic tissue change. Therefore, post-RT interval should be considered a conditional variable whose meaning depends on implant-bed dose, tissue phase, recipient substrate, patient fitness, prosthetic context, and survivor-selection processes, rather than as a universal chronological rule.

## 8. Recipient Site Characteristics and Soft-Tissue Conditions

The risk-adapted concepts proposed in this review apply to implant rehabilitation in both native jaws and reconstructed osseous substrates, but this should not imply biological equivalence or direct transferability of dose–response relationships between recipient sites. Native mandible, native maxilla, vascularized bone flaps, nonvascularized grafts, and surgically altered bone differ in architecture, vascular supply, remodeling potential, soft-tissue coverage, prosthetic accessibility, and exposure to RT dose gradients. Recipient bone type should therefore be considered a modifier of how radiation dose, timing, surgical intervention, and prosthetic loading influence implant outcomes [1,5,15,24,25].

Native mandibular bone may provide favorable primary stability because of its cortical structure, although this advantage can be offset by a high localized RT dose, diminished vascular reserve, mucosal fragility, xerostomia, infection, or proximity to regions at risk for ORN. Native maxillary bone presents different challenges, including lower bone density, sinus proximity, prosthetic biomechanics, and soft-tissue support. Comparisons between mandibular and maxillary implants should therefore account for implant-bed dose, residual bone volume, mucosal condition, prosthetic design, and hygiene accessibility [2,3,4,5,14].

Vascularized bone flaps, particularly fibula free flaps, have expanded implant-supported rehabilitation after segmental mandibular reconstruction. Their vascular supply may provide a favorable biological substrate, and implants placed in fibula bone can support functional rehabilitation in selected patients [1,15,24,25]. Nevertheless, reconstructed bone is not equivalent to native bone; flap height and contour, cortical characteristics, soft-tissue thickness, vestibular depth, implant angulation, prosthetic space, and force distribution may influence outcomes. Biological response may also differ according to whether implants are placed before adjuvant RT, after RT, or in a flap subsequently receiving a high implant-bed dose [5,6,7,8,25].

Peri-implant soft-tissue conditions are equally important. Durable function requires a stable mucosal seal, adequate coverage, hygiene access, and tolerance to prosthetic contact. In HNC survivors treated with RT, fibrosis, mucosal thinning, reduced salivary function, trismus, dysbiosis, scar contracture, flap bulk, and limited vestibular depth may compromise these functions [5,14,18]. Consequently, implant retention alone may not represent treatment success when the surrounding tissues cannot support sustainable prosthetic function [1,13,15].

In reconstructed jaws, no universal soft-tissue thickness or tissue quantity defines an adequate peri-implant environment. The clinically relevant objective is a stable, maintainable tissue configuration that permits appropriate implant emergence and effective hygiene. Excessive flap bulk, scar contracture, or inadequate vestibular depth may require interventions such as skin-paddle thinning or vestibuloplasty. However, available comparative evidence has neither established a flap-specific soft-tissue thickness threshold nor demonstrated a consistent independent effect of skin-paddle tissue type on implant survival [33,34].

Recipient-site characteristics also interact with timing and localized radiation dose. A post-RT implant in low-dose native bone with stable mucosal coverage may have a more favorable biological context than a primary flap implant subsequently exposed to a higher localized dose. Conversely, secondary implantation in reconstructed bone may be reasonable when vascularity, soft-tissue coverage, prosthetic access, and dose are favorable. Recipient substrate should therefore be interpreted within an integrated framework incorporating dose, timing, soft-tissue conditions, and prosthetic feasibility rather than as an isolated variable [5,6,7,8,15,25].

Future studies should report recipient substrate with sufficient granularity, including native mandible or maxilla, vascularized flap bone, nonvascularized grafted bone, or other reconstructed bone. In reconstructed patients, relevant variables include flap type, timing of implantation relative to reconstruction and RT, soft-tissue characteristics or revisions, implant angulation, prosthetic loading, and localized dose. Implant survival should also be distinguished from functional rehabilitation because retained implants in an unfavorable tissue or prosthetic environment may not provide durable patient benefit.

Preventive oral care should remain integral to implant candidacy and long-term rehabilitation. Pretreatment dental and periodontal assessment, control of active infection and disease, professional hygiene, individualized home care, caries prevention, and patient education should be incorporated throughout the HNC treatment pathway; although structured preventive programs are supported, protocols remain heterogeneous [35]. Such measures cannot reverse localized radiation injury but may reduce superimposed inflammatory and infectious burdens.

After RT, xerostomia, fibrosis, trismus, mucosal fragility, altered microbial conditions, and limited hygiene access may compromise peri-implant stability [1,5,14,15,18]. Long-term care should therefore include individualized hygiene instruction, professional maintenance, prevention and early treatment of infection, caries and xerostomia management, periodontal and peri-implant surveillance, and prosthetic designs that permit cleaning [35]. Maintenance intensity should be adapted to salivary function, hygiene access, peri-implant status, prosthetic complexity, implant-bed dose, and overall late-toxicity risk.

Recipient-site assessment should ultimately be multidisciplinary, integrating localized dose history, bone and reconstructive anatomy, prosthetic feasibility and loading, hygiene access, mucosal health, xerostomia, infection risk, and maintenance capacity. Such integration is necessary because identical timing or dose categories may carry different implications according to recipient-site and soft-tissue conditions.

Taken together, recipient bone and peri-implant soft tissues are not passive background variables; they modify how localized radiation injury, surgical trauma, prosthetic loading, and maintenance demands are expressed. Risk-adapted implant rehabilitation after HNC RT must therefore incorporate the anatomical and functional characteristics of the recipient site alongside dose and timing.

## 9. Prosthetic Loading and Functional Rehabilitation Endpoints

Implant survival alone may not capture the goals of oral rehabilitation in HNC survivors treated with RT. Implant-supported rehabilitation should ultimately provide durable, functional, maintainable, and patient-centered benefit. Anatomical changes, irradiated soft tissues, xerostomia, trismus, impaired hygiene access, flap bulk, occlusal instability, and peri-implant inflammation may interfere with the transition from osseointegration to effective prosthetic function [1,5,14,15,24]. Accordingly, implant retention does not necessarily represent successful rehabilitation unless the implant supports a functional, tolerable, and maintainable prosthesis.

Prosthetic loading constitutes an additional biological and mechanical challenge after osseointegration. An initially integrated implant may not withstand long-term loading when peri-implant remodeling capacity is limited, the mucosal seal is fragile, occlusal forces are unfavorable, or hygiene is inadequate. These concerns are particularly relevant after RT, where hypovascularity, hypocellularity, hypoxia, fibroatrophic remodeling, and salivary dysfunction may reduce tissue adaptability to mechanical and microbial stress [5,16,17,18]. Implant survival should therefore be interpreted together with loading status, loading timing, prosthesis type, and continued prosthesis use.

Immediate, early, and conventional loading protocols may have different implications according to implant stability, recipient substrate, soft-tissue quality, implant distribution, occlusion, and hygiene capacity [36,37]. Evidence from general implant populations may not be directly transferable to irradiated HNC survivors. Loading decisions should therefore consider implant-bed dose, tissue quality, reconstructive anatomy, xerostomia, trismus, and late peri-implant risk, and loading protocol should be reported as a biologically relevant variable rather than merely a procedural detail.

Functional rehabilitation is also closely linked to prosthetic design. Fixed and removable implant-supported prostheses differ in force distribution, hygiene access, soft-tissue contact, repairability, and tolerance. In reconstructed mandibles, prosthetic planning may additionally depend on bone height and contour, skin-paddle characteristics, vestibular depth, implant angulation, interarch space, and occlusal relationships [15,24,25]. A retained implant provides limited benefit if the resulting prosthesis is unstable, difficult to maintain, poorly tolerated, or cannot be used because of trismus, xerostomia, or fibrosis. Conversely, fewer well-positioned implants may provide meaningful functional benefit when prosthetic design, hygiene accessibility, and soft-tissue conditions are favorable [1,15,24,25]. Baseline dentition should also be considered because edentulous and partially dentate patients differ in prosthetic objectives, residual dental support, implant requirements, occlusal design, hygiene access, and maintenance needs [1,24,25].

Patient-centered endpoints are therefore essential. Mastication, speech, swallowing, oral competence, comfort, prosthesis use, dietary function, aesthetics, social confidence, and quality of life may provide a more meaningful assessment of rehabilitation than implant survival alone [1,15,24,38]. In a prospective pilot study using the University of Washington Quality of Life questionnaire, Lanzetti et al. reported post-RT deterioration in taste, saliva, and physical-function-related quality-of-life domains [38]. Although not an implant rehabilitation study, these findings reinforce the importance of patient-reported functional outcomes. Biological implant survival should therefore be distinguished from meaningful patient-centered rehabilitation.

Future studies should report prosthetic variables with detail comparable to surgical and dosimetric data [13,15,24,36,37], including whether implants were loaded, timing and protocol of loading, prosthesis type, implant number and distribution, occlusal design, maintenance requirements, complications, and continued prosthesis use. Patient-level functional outcomes and patient-reported measures should also be incorporated where feasible.

In clinical practice, prosthetic loading should follow the same risk-adapted principles as implant placement. Implant-bed dose, recipient substrate, soft-tissue condition, salivary function, trismus, hygiene accessibility, patient factors, and systemic health should inform the loading strategy. In higher-risk irradiated sites, conservative loading, staged rehabilitation, careful occlusal planning, and enhanced maintenance may be appropriate [36,37]. Prosthetic loading is therefore not merely a technical step but a determinant of whether osseointegration translates into durable function.

Overall, implant viability after HNC RT is best viewed as a continuum encompassing placement, osseointegration, loading, prosthesis use, maintenance, function, and complication-free survival. Integrating prosthetic and functional outcomes with implant-bed dosimetry, timing, recipient-site characteristics, and tissue biology provides a more complete assessment of rehabilitation.

## 10. Osteoradionecrosis as an Implant-Relevant Late Toxicity Endpoint

ORN requires specific consideration in implant rehabilitation after head and neck RT. It should be distinguished from routine implant failure, which may result from mechanical, infectious, prosthetic, or osseointegration-related causes that do not meet criteria for ORN. Conversely, ORN should not be treated as entirely independent when it develops anatomically or temporally near implant placement, peri-implant infection, prosthetic trauma, implant removal, or late implant loss [19,39,40]. Both implant failure and ORN may arise from an irradiated tissue bed characterized by reduced vascularity, hypoxia, impaired remodeling, soft-tissue fragility, infection susceptibility, and fibroinflammatory change [14,16,17,18].

Implant placement introduces surgical trauma into tissues with potentially diminished vascular and cellular reserves. Although favorable sites may tolerate this intervention and achieve stable osseointegration, higher-risk sites—particularly those receiving substantial implant-bed doses—may experience trauma or peri-implant inflammation that exceeds local reparative capacity, resulting in delayed healing, bone exposure, infection, peri-implant breakdown, or progressive necrosis [5,6,7,8,14,19,39,40]. Implants therefore do not invariably cause ORN; rather, implant-related events may interact with broader late-toxicity pathways when adverse local factors coexist.

ORN reporting also affects interpretation of implant outcomes. Excluding patients with ORN, severe implant-associated bone loss, major complications, or recurrence may omit clinically important adverse events and overestimate implant survival. Conversely, classifying every ORN event as implant failure is inappropriate because ORN may occur independently of implants and represents a broader spectrum of radiation-related jaw toxicity [14,19,40]. ORN should therefore be reported separately, including its timing, anatomical site, relationship to implant placement or loading, and localized radiation dose.

Long-term follow-up is essential because early osseointegration may be demonstrated within months, whereas ORN, late peri-implant breakdown, marginal bone loss, and prosthesis-related trauma may emerge years after RT. Short follow-up can therefore document early retention while missing clinically important late toxicities, particularly in high-dose mandibular or reconstructed sites with reduced vascular reserve, mucosal integrity, hygiene access, or prosthetic tolerance [5,6,7,8,14,19].

Future studies should prospectively define ORN as a distinct implant-relevant late toxicity endpoint and document its timing relative to implant placement, loading, peri-implant infection, or removal [19,39,40]. Reporting should include implant-bed dose, anatomical site, substrate type, relevant prior surgery or extractions, prosthetic trauma, infection, management, and the relationship between implant loss and ORN. Such information would distinguish routine implant failure from severe radiation-related jaw toxicity while preserving their clinically relevant association.

Clinically, ORN risk should guide individualized implant planning rather than function as a universal contraindication. Risk reduction should emphasize candidate and site selection, implant-bed-specific radiation exposure, infection control, soft-tissue integrity, oral hygiene and modifiable host factors, and appropriately conservative surgical and prosthetic planning [5,6,7,8,14,19,41]. Patients with lower implant-bed doses, favorable mucosal coverage, adequate hygiene access, stable oncologic status, and manageable prosthetic requirements may remain suitable candidates, whereas high-dose mandibular exposure, prior ORN, active infection, compromised mucosa, severe xerostomia, repeated surgery, or complex prosthetic needs may justify more conservative or staged approaches [5,6,7,8,14,19].

Adjunctive interventions should be interpreted according to their indication and evidence base. Hyperbaric oxygen (HBO) has historically been used in irradiated jaws, but the HOPON randomized trial found no reduction in ORN with prophylactic HBO among patients undergoing extraction or implant placement in mandibular regions exposed to >50 Gy [42]. Consistently, the 2024 ISOO–MASCC–ASCO guideline does not recommend routine prophylactic HBO before invasive dental procedures after head and neck RT, although selective use may be considered in high-risk situations [41]. HBO should therefore not substitute for implant-bed-specific dosimetric and tissue assessment.

Pentoxifylline and tocopherol (PENTO) have also been investigated for modulation of radiation-induced fibroatrophy. Although observational studies have reported favorable outcomes, a systematic review found insufficient evidence to establish prophylactic efficacy because of limited and heterogeneous nonrandomized data [43]. The 2024 ISOO–MASCC–ASCO guideline provides only a weak recommendation, based on low-quality evidence, for perioperative PENTO in selected cancer-free patients at elevated ORN risk undergoing invasive dental procedures [41]. Importantly, the evidence derives predominantly from extraction cohorts and does not establish improved implant osseointegration or survival. Addition of clodronate (PENTOCLO) has primarily been investigated for treatment of established ORN, with randomized evidence still developing, including the RAPTOR trial [44]. Adjunctive therapies may therefore have selected roles but should not replace assessment of localized dose, recipient substrate, soft-tissue integrity, infection, and prosthetic feasibility.

Accordingly, ORN should be regarded neither as synonymous with implant failure nor as an extraneous exclusion. It is an implant-relevant, time-dependent late toxicity endpoint that should be reported alongside implant survival, peri-implant bone status, prosthetic outcomes, and patient-centered rehabilitation measures.

## 11. Methodological Pitfalls in the Existing Literature

Biological heterogeneity and recurring limitations in study design, outcome definition, exposure measurement, and statistical analysis complicate interpretation of dental implant outcomes after head and neck RT. Much of the evidence is retrospective, heterogeneous, and based on relatively small cohorts with multiple implants per patient; apparently precise implant-level estimates may therefore not translate into reliable patient-level inference.

A central issue is intrapatient clustering. Multiple implants within one patient cannot be assumed to represent independent observations because they share systemic factors, oncologic treatment, radiation exposure, oral environment, reconstructive status, prosthetic loading, and follow-up. Failure to account for clustering can underestimate standard errors and overstate precision. This problem is recognized in dental research [45], and implant failures themselves may cluster within patients. Appropriate approaches include generalized estimating equations, mixed-effects models, frailty or marginal survival models, and cluster-robust standard errors [46,47].

Outcome definitions are also inconsistent. Implant survival, success, osseointegration, prosthetic loading, functional rehabilitation, peri-implant bone stability, and ORN are frequently used inconsistently or collapsed into binary endpoints. This may overestimate clinical benefit when retained implants have limited prosthetic use, impaired function, recurrent inflammation, or late toxicity [11,12,13,14,15]. Biological integration, implant retention, prosthetic success, patient-level rehabilitation, and long-term complication-free viability should therefore be distinguished.

Measurement of RT exposure represents another major limitation. Prescribed tumor dose, irradiated versus non-irradiated classifications, and broad dose categories may misclassify the biologically relevant exposure because actual dose varies substantially across implant sites [5,6,7,8]. Physical dose may also not be directly comparable across different fractionation schedules, boosts, or reirradiation [20,21]. Future studies should therefore report implant-bed-specific dose metrics, relevant dose-volume parameters, fractionation, and EQD2 when appropriate.

Timing variables are likewise often modeled inadequately. Pre- versus post-RT placement, immediate versus delayed placement, and post-RT intervals are frequently treated categorically despite potentially nonlinear and context-dependent effects [9,10,27,28]. Arbitrary thresholds such as 6, 12, 18, or 24 months should not be presumed to represent biological recovery. Where feasible, continuous modeling with restricted cubic splines, fractional polynomials, segmented regression, or other flexible approaches is preferable [29,30], with interaction analyses used to examine modification by implant-bed dose, recipient substrate, soft-tissue conditions, or prosthetic loading.

Selection bias and confounding by indication are particularly relevant in delayed post-RT cohorts. Patients implanted years after RT are often recurrence-free, medically fit, and judged suitable for elective rehabilitation, whereas those with recurrence, severe toxicity, poor healing, ORN, or other adverse conditions may never receive implants. Apparent benefits of delayed implantation may therefore partly reflect selection of healthier survivors [31,32]. Eligibility criteria, reasons for non-placement, post-RT exclusions, and baseline differences should be reported transparently.

Follow-up duration also affects interpretation. Short-term studies may document osseointegration while missing late implant loss, prosthetic non-use, marginal bone deterioration, peri-implant infection, ORN, or impaired functional rehabilitation. Because such complications may emerge years after RT, short or incomplete follow-up can underestimate adverse outcomes [14,19,39,40]. Time-to-event analyses are preferable when follow-up varies and should distinguish early integration from long-term complication-free viability.

Competing events further complicate post-RT rehabilitation. Recurrence, death, medical deterioration, ORN, severe toxicity, or inability to proceed to loading may preclude implantation or functional rehabilitation. Analyses restricted to patients who ultimately receive implants may therefore omit important portions of the rehabilitation pathway, particularly when longer post-RT intervals inherently require continued survival and clinical fitness. Studies should document the pathway from referral and eligibility assessment through implant placement, loading, prosthesis use, complications, and discontinuation.

The principal methodological limitations, their potential consequences, and recommended reporting or analytic approaches are summarized in Table 3.

In summary, future studies should move beyond simple implant-level survival estimates and categorical timing comparisons. Implant-bed-specific dosimetry, clearly defined outcomes, continuous timing models, cluster-adjusted analyses, time-to-event methods, and transparent reporting of selection pathways should improve the ability to distinguish true determinants of implant viability from exposure misclassification, survivor selection, and analytic artifacts.

## 12. Toward a Risk-Adapted Framework for Implant Rehabilitation After HNC RT

The preceding sections suggest that dental implant viability following head and neck RT is not determined by any single variable—such as RT exposure, prescribed dose, placement timing, or post-treatment interval. A more informative approach is to view implant rehabilitation as a risk-adapted process shaped by the interplay among localized radiation exposure, tissue biology, recipient substrate, prosthetic loading, systemic factors, and methodological aspects of outcome assessment. This framework supports a shift from fixed chronological rules toward individualized evaluation of the implant bed.

Within this framework, implant-bed-specific dose should be interpreted as a graded risk variable rather than a fixed threshold, with localized exposure considered in its fractionation, boost, reirradiation, and anatomical context [5,6,7,8,20,21]. Timing should likewise be regarded as a contextual modifier rather than an independent determinant: the potential advantages of pre-RT or post-RT placement depend on the subsequent or preceding implant-bed dose, tissue condition, and prosthetic environment [6,7,8,24,25,26].

The recipient substrate and soft-tissue conditions constitute a second major domain for risk stratification. Native mandible, native maxilla, vascularized bone flaps, nonvascularized grafts, and surgically altered bone differ in vascularity, cortical structure, remodeling capacity, prosthetic access, and response to RT [1,15,24,25]. In addition, factors such as fibrosis, xerostomia, mucosal thinning, trismus, flap bulk, limited vestibular depth, dysbiosis, and reduced access for hygiene may compromise long-term peri-implant stability, even after initial osseointegration is achieved [5,14,18]. Comprehensive substrate assessment should therefore address both bone and soft tissue, as implant viability relies on the integration of the implant–bone–mucosa–prosthesis interface.

Prosthetic feasibility is a third essential domain. Evaluation should consider not only implant retention but also the ability to support a functional, tolerable, maintainable, and patient-centered prosthesis [1,13,15]. Variables such as loading protocol, prosthesis design, number and distribution of implants, occlusal scheme, interarch space, accessibility for hygiene, maintenance requirements, and ongoing prosthesis use contribute to risk assessment [36,37]. In high-risk irradiated sites, approaches such as conservative loading, staged rehabilitation, careful occlusal planning, and enhanced maintenance may be preferable to rapid or excessive functional loading.

This framework should incorporate systemic and behavioral factors. Smoking, diabetes, malnutrition, vascular disease, immunosuppression, suboptimal oral hygiene, alcohol use, limited access to specialized follow-up, and inconsistent maintenance may reduce the likelihood that osseointegration will result in durable rehabilitation. These factors may also interact with RT dose and tissue substrate; for example, a borderline implant-bed dose may be acceptable in a motivated patient with excellent hygiene and stable soft tissues but is associated with higher risk in individuals with xerostomia, tobacco use, poor nutrition, or limited maintenance capacity.

Consider late toxicity risk, particularly ORN, within a risk-adapted framework, without conflating ORN with routine implant failure. ORN is best regarded as an implant-relevant late toxicity endpoint when it occurs temporally or anatomically in relation to implant placement, peri-implant infection, prosthetic trauma, implant removal, or late implant loss [19,39,40]. In this context, factors such as prior ORN, high-dose mandibular exposure, active infection, exposed bone, repeated surgical intervention, or compromised mucosal integrity should prompt consideration of more conservative or alternative rehabilitation strategies.

Together, these considerations define six principal domains for risk-adapted assessment: implant-bed dose, timing relative to RT, recipient substrate, soft-tissue conditions, prosthetic feasibility, and host or late-toxicity risk. Table 4 summarizes the relative strength, directness, and principal limitations of the evidence supporting these domains. Because this article is a critical narrative review and did not include a formal systematic risk-of-bias or certainty-of-evidence assessment, do not interpret these qualitative categories as GRADE or equivalent evidence ratings.

In routine clinical practice, implementation can follow a sequential multidisciplinary assessment. First, anatomically define the proposed implant site and establish its localized RT exposure from treatment-planning data when available [5,6,7,8,20,21]. Second, recipient bone and peri-implant tissue conditions—including substrate type, mucosal integrity, xerostomia, fibrosis, infection, trismus, and hygiene accessibility—should be assessed [1,5,14,15,24,25]. Third, prosthetic feasibility should be established with consideration of implant position, anticipated loading, occlusal demands, hygiene access, and maintainability [1,13,15,36,37]. Finally, incorporate host, oncologic, and late-toxicity factors into the overall assessment [5,14,19]. The resulting profile may support individualized implant planning, indicate the need for optimization or enhanced surveillance, or favor deferral or an alternative rehabilitation strategy. When rehabilitation is anticipated before RT, potential implant-bearing regions may be considered during treatment planning; after RT, localized exposure can be reconstructed from the original treatment plan. This workflow is intended to structure multidisciplinary clinical reasoning while preserving oncologic priorities rather than to generate a numerical risk score.

**Figure 3:**
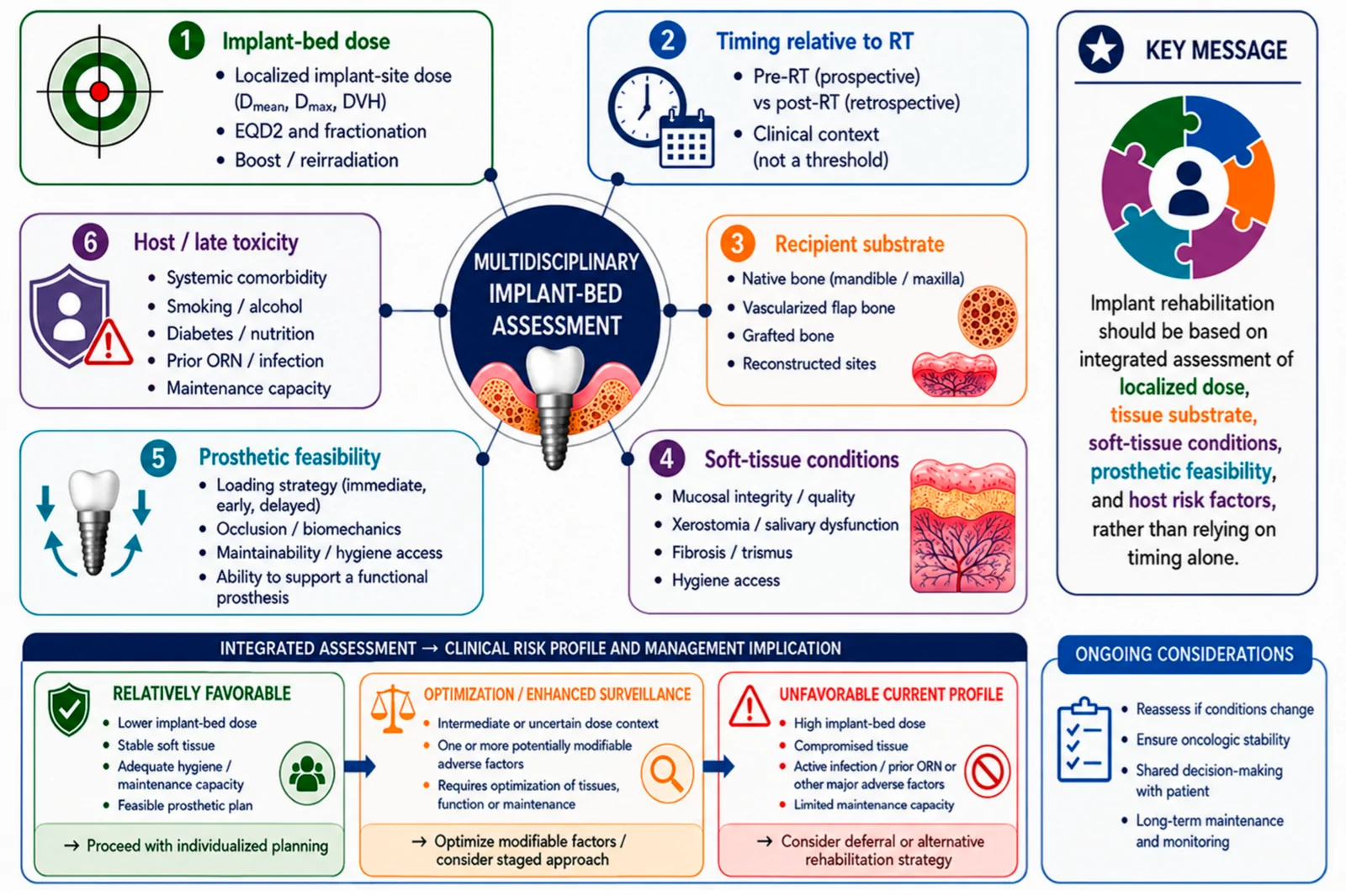
Practical Risk-Adapted Framework for Dental Implant Rehabilitation After Head and Neck Radiotherapy. The proposed framework integrates six domains relevant to implant rehabilitation: implant-bed radiation dose, timing relative to RT, recipient substrate, soft-tissue conditions, prosthetic feasibility, and host/late-toxicity factors. Their combined assessment is used to characterize the clinical situation as relatively favorable, requiring optimization or enhanced surveillance, or unfavorable for current implantation, with corresponding implications for individualized planning, optimization of modifiable factors, deferral, or alternative rehabilitation strategies. These categories represent qualitative clinical profiles rather than validated risk strata and should not be interpreted as numerical probability estimates or deterministic eligibility criteria. The framework is intended to support multidisciplinary clinical reasoning while preserving oncologic priorities and individualized patient assessment. Abbreviations: Dmean, mean dose; Dmax, maximum dose; DVH, dose–volume histogram; EQD2, equivalent dose in 2-Gy fractions; ORN, osteoradionecrosis; RT, radiotherapy.

The framework also emphasizes interactions among risk factors rather than isolated predictors. Future research should determine whether combinations—such as a high implant-bed dose with unfavorable soft-tissue conditions or a favorable dose with optimized prosthetic loading—better predict long-term rehabilitation than timing or prescribed dose alone. Minimum reporting domains for such studies are summarized in Table 5, while the practical decision pathway is illustrated in Figure 3.

Ultimately, implementing risk-adapted implant rehabilitation following HNC RT requires multidisciplinary coordination. Radiation oncologists can contribute localized dose data; surgeons assess bone and reconstructive anatomy; prosthodontists evaluate loading strategies, prosthesis design, and maintainability; and dental oncologists manage oral hygiene, mucosal health, xerostomia, infection, and ongoing surveillance. This collaborative approach shifts implant planning from a chronological decision to a biologically informed survivorship strategy.

## 13. Future Research and Reporting Standards

Future research on dental implant rehabilitation after head and neck RT should move beyond isolated survival estimates toward standardized reporting that reflects the biological, dosimetric, prosthetic, and methodological complexity of this population. Although implant-supported rehabilitation is feasible in selected irradiated patients, interpretation remains limited by heterogeneous endpoints, non-localized dose reporting, arbitrary timing and dose categories, inadequate handling of multiple implants per patient, and variable follow-up [2,3,4,9,10,15]. Future studies should therefore define the conditions under which implants provide durable functional rehabilitation rather than simply whether they remain in situ.

First, radiation exposure should be reported at the implant-bed level whenever possible. Prescribed tumor dose should be distinguished from mandibular, maxillary, reconstructed-bone, and implant-bed-specific exposure, with implant-site Dmean, Dmax, and relevant dose-volume parameters extracted when available [5,6,7,8]. Fraction size, number of fractions, boost or reirradiation, and EQD2 should also be reported when relevant [20,21].

Dosimetric variables should preferably be analyzed continuously rather than primarily dichotomized using data-derived thresholds. Flexible approaches such as restricted cubic splines, fractional polynomials, or segmented regression can evaluate graded or nonlinear dose–response relationships, while proposed thresholds should remain exploratory unless externally validated or biologically justified [29,30].

Second, timing should be reported precisely, including placement relative to RT, ablative or reconstructive surgery, and prosthetic loading [9,10,24,25,26]. For post-RT implantation, the interval from RT completion to placement should preferably remain continuous; flexible modeling may identify nonlinear relationships [27,28]. Timing should be interpreted together with implant-bed dose, recipient substrate, soft-tissue conditions, and prosthetic loading rather than as an isolated determinant.

Third, outcomes should distinguish early osseointegration, implant retention, implant success, prosthetic loading and use, peri-implant bone status, infection, late implant loss, ORN, and patient-centered functional outcomes [11,12,13,14,15,19]. Implant survival should not be equated with successful rehabilitation when a retained implant cannot support a functional and maintainable prosthesis or meaningful patient benefit [1,13,15,24].

Fourth, recipient-site reporting should distinguish native mandible, native maxilla, vascularized flap bone, nonvascularized grafts, and other reconstructed substrates, with relevant reconstructive and prosthetic characteristics documented [1,15,24,25]. Soft-tissue and functional variables—including xerostomia, mucosal integrity, fibrosis, trismus, hygiene access, salivary function, and peri-implant stability—should also be recorded [5,14,18].

Fifth, prosthetic variables should be treated as substantive outcomes. Loading status and timing, prosthesis type, supporting implant distribution, occlusal design, complications, maintenance, and continued prosthesis use should be reported [36,37]. Functional and patient-reported outcomes should be incorporated when feasible because the ultimate objective is improvement in oral function, comfort, dietary capacity, social participation, and quality of life [1,15,24].

Sixth, analyses must account for the hierarchical structure of implant data. Multiple implants within one patient should not be treated as independent observations; generalized estimating equations, mixed-effects models, frailty or marginal survival models, or cluster-robust standard errors are appropriate options depending on the endpoint and design [45,46,47]. Implant-level and patient-level outcomes should also be distinguished.

Seventh, studies should explicitly address selection bias, confounding by indication, and competing events. Eligibility and referral pathways, reasons for non-placement, post-RT exclusions, recurrence, medical ineligibility, prior ORN, severe toxicity, and failure to proceed to loading or rehabilitation should be documented [31,32]. Time-to-event analyses should account for variable follow-up and clinically relevant competing events, particularly in delayed post-RT cohorts in which longer intervals inherently require continued survival and clinical fitness.

Finally, ORN should be reported as a distinct implant-relevant late toxicity endpoint, including its timing relative to implant placement, loading, infection, or removal [19,39,40]. Anatomical relationship to the implant bed, localized dose, infection or prosthetic trauma, management, and association with implant loss should be documented to distinguish routine implant failure from severe radiation-associated jaw toxicity.

Adoption of these standards would enable future studies to progress from descriptive implant survival toward biologically and clinically interpretable risk stratification. Ideally, future evidence should derive from prospective, multicenter, dosimetry-integrated studies adequately powered to examine interactions among implant-bed dose, timing, recipient substrate, soft-tissue conditions, prosthetic loading, and host factors. Until then, transparent reporting and bias-aware analysis remain essential for individualized rehabilitation after HNC RT.

## 14. Limitations of the Review

Several limitations of the present review should be acknowledged. As a critical narrative review, it did not employ a formal systematic-review methodology, prespecified study-selection process, quantitative evidence synthesis, or structured risk-of-bias assessment. Consequently, despite the use of a structured literature search and reference screening, the selection and relative emphasis of the evidence discussed may be susceptible to selection bias and necessarily involve interpretive judgment. The review should therefore not be regarded as an exhaustive evidence synthesis or formal assessment of certainty of evidence. Rather, its purpose is to critically examine recurring conceptual and methodological limitations in the literature and to propose a biologically and clinically informed framework for future research, reporting, and implant rehabilitation after head and neck radiotherapy.

## 15. Conclusions

Dental implant-supported rehabilitation after head and neck RT should no longer be interpreted primarily through simplified chronological rules or broad radiation categories. Conventional constructs such as irradiated versus non-irradiated status, pre-RT versus post-RT placement, prescribed dose, and post-treatment waiting interval remain clinically convenient, but they do not fully capture the biological conditions that determine long-term implant viability. The central exposure is the localized radiation dose delivered to the implant bed, ideally interpreted together with fractionation, recipient substrate, soft-tissue quality, prosthetic loading, systemic host factors, and late toxicity risk.

A biologically informed interpretation of implant outcomes requires a clear distinction among early osseointegration, implant retention, implant success, prosthetic rehabilitation, and patient-centered functional benefit. In irradiated HNC survivors, a retained implant is not necessarily a successful rehabilitation outcome if it cannot support a tolerable, maintainable, and functional prosthesis or if it is accompanied by late peri-implant complications. Similarly, longer waiting after RT should not be assumed to indicate progressive biological recovery, because chronic hypovascular, hypocellular, hypoxic, and fibroatrophic tissue states may persist despite apparent clinical stability.

The available literature, therefore, supports a shift from timing-centered decision-making toward implant-bed-specific, risk-adapted rehabilitation planning. Future research should integrate localized dosimetry, continuous assessment of dose and timing, standardized outcome definitions, cluster-aware statistical methods, time-to-event endpoints, and patient-level functional outcomes. ORN should be reported as a distinct implant-relevant late toxicity endpoint rather than conflated with routine implant failure or excluded from interpretation.

Ultimately, safe and meaningful implant rehabilitation after HNC RT requires coordinated input from radiation oncologists, surgeons, prosthodontists, dental oncologists, and survivorship teams. The key clinical question is not simply when an implant is placed, but whether the specific implant bed retains sufficient biological, anatomical, prosthetic, and patient-related capacity to support durable function. Moving from chronological waiting rules to implant-bed-specific risk stratification offers a more precise framework for research, reporting, and individualized clinical decision-making.

## Figures and Tables

**Table 1:** Proposed outcome hierarchy for dental implant rehabilitation after head and neck radiotherapy.

Outcome Domain	Working Definition	Clinical Meaning	Key Limitation If Reported Alone
Early osseointegration	Initial biological anchorage of the implant within bone, usually assessed during early healing	Indicates early recipient-site compatibility	Does not prove long-term stability in irradiated tissue
Implant survival	Implant remains in situ, regardless of symptoms, loading, function, or complications	Basic retention endpoint	May overestimate clinical success
Implant success	Retained implant without mobility, pain, infection, progressive bone loss, or peri-implant pathology	More stringent biological and clinical endpoint	May still not capture prosthetic use or patient benefit
Prosthetic rehabilitation success	Implant supports a functional, tolerable, maintainable prosthesis	Reflects clinically meaningful rehabilitation	Requires reporting of loading, prosthesis use, complications, and maintenance
Patient-centered functional benefit	Improvement in mastication, speech, swallowing, comfort, aesthetics, diet, social confidence, or quality of life	Captures survivorship value	Often underreported or inconsistently measured
Osteoradionecrosis as late toxicity endpoint	Osteoradionecrosis reported separately when temporally or anatomically related to implant treatment	Identifies severe radiation-associated jaw toxicity relevant to implant safety	Should not be conflated with routine implant failure

Note: The proposed hierarchy distinguishes progressively broader dimensions of implant rehabilitation, from early biological integration to patient-centered functional benefit and late toxicity. The working definitions are intended to improve conceptual and reporting clarity and should not be interpreted as validated composite criteria or a scoring system.

**Table 2:** Radiobiological effects of head and neck radiotherapy relevant to implant rehabilitation.

Tissue Compartment	Radiation-Induced Effects	Potential Consequences for Implant Rehabilitation
Bone	Osteoblast depletion, reduced osteoprogenitor activity, impaired remodeling, osteocyte injury	Delayed osseointegration, marginal bone loss, reduced adaptive remodeling, implant instability, late implant failure
Microvasculature	Endothelial dysfunction, capillary rarefaction, luminal narrowing, thrombosis, reduced perfusion	Impaired wound healing, reduced oxygen and nutrient delivery, increased susceptibility to tissue breakdown
Connective tissue/stroma	Fibroatrophic remodeling, extracellular matrix deposition, chronic fibrosis	Reduced tissue elasticity, diminished stress tolerance, impaired long-term tissue adaptation
Mucosa and peri-implant soft tissue	Mucosal thinning, impaired regenerative capacity, tissue fragility	Compromised soft-tissue coverage and peri-implant mucosal seal, soft-tissue breakdown, reduced tolerance to surgical and prosthetic trauma, and increased peri-implant inflammation
Salivary glands	Reduced salivary secretion, xerostomia, and altered oral environment	Increased microbial burden, impaired hygiene maintenance, and increased peri-implant inflammation
Immune and inflammatory pathways	Persistent inflammatory signaling, oxidative stress, and altered host defense mechanisms	Delayed tissue repair, infection susceptibility, and chronic peri-implant inflammatory changes
Composite tissue response	Hypovascularity, hypocellularity, hypoxia, fibroinflammatory remodeling	Increased vulnerability to late implant failure and osteoradionecrosis

Note: The listed consequences summarize biologically and clinically relevant implications of radiation-associated tissue injury for implant rehabilitation. They represent a conceptual synthesis and should not be interpreted as quantified effect estimates or deterministic outcomes.

**Table 3:** Methodological limitations in studies of dental implant outcomes after head and neck radiotherapy.

Methodological Limitation	Potential Impact on Interpretation	Recommended Approach
Non-localized RT exposure reporting	Prescribed tumor dose or broad irradiated/non-irradiated categories may misclassify the biologically relevant implant-bed exposure	Report implant-bed-specific Dmean, Dmax, relevant dose-volume metrics, fractionation, boost/reirradiation status, and EQD2 when appropriate
Data-driven dose cutoffs	Arbitrary or cohort-derived thresholds may create artificial risk groups and obscure graded dose–response relationships	Analyze dosimetric variables continuously using flexible models; treat thresholds as exploratory unless externally validated
Arbitrary timing categories	Cutoffs such as 6, 12, 18, or 24 months may not correspond to true biological recovery phases	Report post-RT interval continuously and assess nonlinear timing effects where feasible
Implant-level clustering	Multiple implants within one patient share biological, treatment, prosthetic, and follow-up characteristics	Use generalized estimating equations, mixed-effects models, frailty models, marginal survival models, or cluster-robust standard errors
Inconsistent outcome definitions	Implant survival may be conflated with success, prosthetic function, or patient-level rehabilitation	Separately report osseointegration, implant retention, implant success, prosthetic loading/use, functional outcomes, and late toxicity
Short or variable follow-up	Early osseointegration may be captured while late implant loss, ORN, peri-implant bone loss, or prosthetic failure are missed	Use time-to-event analyses and distinguish early integration from late complication-free viability
Survivor-selection bias	Delayed post-RT implant cohorts may overrepresent recurrence-free, medically fit, lower-risk survivors	Report eligibility pathways, reasons for non-placement, exclusions after RT, recurrence status, and baseline differences
Confounding by indication	Implant timing may reflect clinician selection based on tissue quality, dose, prognosis, or patient fitness	Adjust for clinically relevant confounders and interpret timing effects as potentially selected rather than causal
Competing events	Recurrence, death, ORN, medical deterioration, or inability to proceed to loading may preclude implant rehabilitation	Document competing events and analyze the full rehabilitation pathway from referral to prosthetic use
Limited prosthetic reporting	Retained implants may not translate into functional rehabilitation if loading, prosthesis use, or maintenance are unreported	Report loading protocol, prosthesis type, active use, complications, maintenance burden, and patient-reported benefit

Note: Recommended approaches indicate reporting or analytic strategies intended to reduce the corresponding methodological limitations; they should not be interpreted as formal consensus standards. Abbreviations: Dmean, mean dose; Dmax, maximum dose; EQD2, equivalent dose in 2-Gy fractions; ORN, osteoradionecrosis; RT, radiotherapy.

**Table 4:** Qualitative summary of risk factors relevant to dental implant rehabilitation after head and neck radiotherapy.

Risk Factor/Domain	Qualitative Evidence Strength	Interpretation	Principal Limitations	Key References
Implant-bed-specific RT dose	Relatively stronger	Localized implant-bed dose shows the most consistent implant-specific dose–response signal; lower-dose regions appear more favorable, whereas risk increases with greater localized exposure.	Predominantly retrospective cohorts; relatively small samples; heterogeneous endpoints and RT techniques; no externally validated universal cutoff.	[6,7,8,22]
Timing relative to RT	Moderate but inconsistent	Timing may influence outcomes, but evidence does not establish a universally optimal post-RT waiting interval independent of local dose and tissue condition.	Heterogeneous and often arbitrary timing categories; confounding by indication; survivor-selection bias; limited adjustment for implant-bed dose.	[9,10,27,28]
Anatomical implant location	Moderate	Mandibular versus maxillary location may influence risk through differences in bone structure, vascularity, biomechanics, and radiation exposure, but location should not be interpreted independently of local dose and substrate.	Anatomical site is frequently confounded by dose distribution, tumor location, surgery, and reconstructive status.	[2,3,4,5,19]
Recipient bone quality and substrate	Moderate	Native mandible, native maxilla, vascularized flap bone, grafted bone, and reconstructed sites differ biologically and mechanically and should be assessed separately.	Substrate definitions and bone-quality measures are inconsistent; reconstructed cohorts are heterogeneous; relatively few comparative studies.	[1,15,24,25]
Soft-tissue environment, xerostomia, and hygiene capacity	Limited/indirect	Mucosal integrity, fibrosis, salivary dysfunction, trismus, and hygiene accessibility are clinically relevant modifiers of peri-implant maintenance and functional rehabilitation.	Much of the evidence is biological, ORN-related, or extrapolated from broader HNC survivorship literature rather than implant-specific comparative studies.	[5,14,18]
Prosthetic loading and biomechanics	Limited/indirect in irradiated HNC populations	Loading strategy, prosthetic design, occlusal forces, implant distribution, and maintainability plausibly modify long-term rehabilitation success.	Much of the evidence derives from general implant populations; limited direct comparative evidence in irradiated HNC survivors.	[1,15,24,36,37]
Systemic and behavioral factors	Limited/heterogeneous in irradiated HNC populations	Smoking, diabetes, nutritional status, vascular disease, immunosuppression, oral hygiene, and maintenance capacity may modify implant risk and interact with local tissue conditions.	Individual factors are inconsistently reported and adjusted for; HNC-specific implant evidence is sparse; substantial potential for confounding.	[1,5,15,47]
Prior ORN, active infection, and compromised local tissue integrity	Moderate for clinical relevance; limited for quantitative prediction	These features identify a clinically unfavorable tissue environment and warrant more conservative individualized assessment.	ORN definitions and attribution vary; implant-associated and spontaneous ORN may be conflated; quantitative implant-specific risk estimates remain limited.	[19,39,40]

Note: Evidence strength is qualitative and reflects the relative directness, consistency, and methodological depth of the literature reviewed; it does not represent a formal certainty-of-evidence assessment using the Grading of Recommendations Assessment, Development and Evaluation framework or another systematic approach. “Relatively stronger” denotes comparatively more consistent implant-specific evidence within an evidence base that remains predominantly observational. Abbreviations: HNC, head and neck cancer; ORN, osteoradionecrosis; RT, radiotherapy.

**Table 5:** Proposed reporting standards for future studies of dental implants after head and neck radiotherapy.

Domain	Minimum Reporting Recommendations
Study population	HNC subsite, tumor stage where available, treatment intent, RT modality, systemic therapy, surgery/reconstruction status, baseline dentition status (fully edentulous or partially dentate), recurrence status, eligibility criteria, and reasons for non-placement or exclusion
Implant-bed dosimetry	Prescribed tumor dose distinguished from mandibular, maxillary, reconstructed-bone, and implant-bed-specific dose; implant-site Dmean, Dmax, relevant dose-volume metrics, boost/reirradiation status, fractionation, and EQD2 where appropriate
Timing variables	Pre-RT, intraoperative/primary, post-RT, or delayed placement; timing relative to reconstruction, RT completion, loading, and prosthetic delivery; post-RT interval reported continuously
Recipient substrate	Native mandible, native maxilla, vascularized bone flap, nonvascularized graft, previously reconstructed bone; flap type, flap irradiation status, implant angulation, interarch space, vestibuloplasty or soft-tissue revision
Soft-tissue environment	Mucosal integrity, fibrosis, xerostomia, trismus, salivary function, hygiene accessibility, soft-tissue coverage, peri-implant mucosal stability, infection status
Prosthetic rehabilitation	Loading status, time to loading, loading protocol, prosthesis type, number and distribution of supporting implants, occlusal scheme, active prosthesis use, complications, maintenance requirements
Outcome definitions	Early osseointegration, implant retention, implant success, peri-implant bone stability, peri-implant infection, late implant loss, prosthetic use, functional rehabilitation, patient-reported outcomes
ORN reporting	ORN before or after implant placement, relationship to loading/infection/removal, anatomical proximity to implant bed, localized dose context, management required, association with implant loss
Statistical analysis	Patient-level and implant-level outcomes distinguished; clustering addressed when multiple implants are analyzed; dose and timing modeled continuously where feasible; time-to-event methods used when follow-up varies
Selection and competing events	Referral pathway, eligibility assessment, reasons for non-placement, post-RT exclusions, recurrence, death, medical ineligibility, severe toxicity, ORN, failure to proceed to loading or prosthetic rehabilitation

Note: These proposed minimum reporting domains are intended to improve transparency, comparability, and interpretability across future studies and should not be interpreted as a validated reporting checklist or formal consensus guideline. Abbreviations: Dmean, mean dose; Dmax, maximum dose; EQD2, equivalent dose in 2-Gy fractions; HNC, head and neck cancer; ORN, osteoradionecrosis; RT, radiotherapy.

## Data Availability

No new data were created or analyzed in this study. Data sharing is not applicable to this article.
